# An ultrathin, rapidly fabricated, flexible giant magnetoresistive electronic skin

**DOI:** 10.1038/s41378-024-00716-2

**Published:** 2024-08-12

**Authors:** Junjie Zhang, Zhenhu Jin, Guangyuan Chen, Jiamin Chen

**Affiliations:** 1 State Key Laboratory of Transducer Technology, Aerospace Information Research Institute, Chinese Academy of Sciences, Beijing, 100190 China; 2 School of Electronic, Electrical and Communication Engineering, University of Chinese Academy of Sciences, Beijing, 100049 China; 3 College of Materials Sciences and Opto-Electronic Technology, University of Chinese Academy of Sciences, Beijing, 100049 China

**Keywords:** Sensors, Electrical and electronic engineering, Electronic devices

## Abstract

In recent years, there has been a significant increase in the prevalence of electronic wearables, among which flexible magnetoelectronic skin has emerged as a key component. This technology is part of the rapidly progressing field of flexible wearable electronics, which has facilitated a new human perceptual development known as the magnetic sense. However, the magnetoelectronic skin is limited due to its low sensitivity and substantial field limitations as a wearable electronic device for sensing minor magnetic fields. Additionally, achieving efficient and non-destructive delamination in flexible magnetic sensors remains a significant challenge, hindering their development. In this study, we demonstrate a novel magnetoelectronic touchless interactive device that utilizes a flexible giant magnetoresistive sensor array. The flexible magnetic sensor array was developed through an electrochemical delamination process, and the resultant ultra-thin flexible electronic system possessed both ultra-thin and non-destructive characteristics. The flexible magnetic sensor is capable of achieving a bending angle of up to 90 degrees, maintaining its performance integrity even after multiple repetitive bending cycles. Our study also provides demonstrations of non-contact interaction and pressure sensing. This research is anticipated to significantly contribute to the advancement of high-performance flexible magnetic sensors and catalyze the development of more sophisticated magnetic electronic skins.

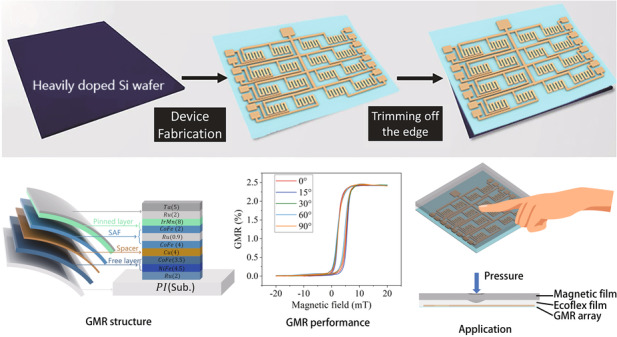

## Introduction

Over the past two decades, the prevalence of electronic wearables has drastically increased, particularly in the domain of human-to-machine interfaces (HMIs)^1–3^. Electronic devices that monitor human health, track movement and activities, and function as HMIs are already in widespread use^1,2,4^. However, the goal of these electronic skins is to emulate the features of skin while still preserving its natural qualities^5–7^. Flexible magnetic sensors, such as giant magnetoresistive (GMR)^8–21^, tunneling magnetoresistive (TMR)^22–24^, anisotropic magnetoresistive (AMR)^25,26^, giant magnetoimpedance (GMI)^27^, and Hall^28,29^ sensors, have become critical components of this rapidly evolving HMI field. These flexible magnetic sensors facilitate a new path in human perceptual development since they have the potential to form a new perception, known as magnetic perception^30,31^.

Giant magnetoresistance (GMR) sensors have become predominant in magnetic detection due to their significant variations in electrical resistance^8,14,15^. Currently, flexible GMR sensors are primarily fabricated through the deposition of Co/Cu^17,21,28^, Py/Cu^19,21,32^, or Pd/Co^15^ stacks onto ultrathin foils. However, this configuration exhibits limited sensitivity to small magnetic fields. Furthermore, the conventional fabrication of flexible GMR sensors requires introducing a sacrificial layer between the polymer substrate and the rigid support^33^. The separation process is often manual, inefficient, and complicates mass production. Moreover, existing GMR sensors have not yet exploited the benefits provided by array sensor configurations.

In this study, a novel magnetoelectronic, touchless interactive system utilizing a flexible giant magnetoresistive (GMR) sensor array, sensitive to small magnetic fields, was developed. A flexible magnetic sensor array was fabricated using an electrochemical delamination process, enabling the successful detachment of the ultrathin films from a rigid substrate without performance degradation. This delamination process was efficient and rapid and caused no damage to the electronic system. The adapted GMR spin valve structure provided an effective field of below 80 Oe. Flexible GMR sensors are capable of achieving a bending angle of 90° and exhibit durability against multiple instances of repetitive bending. Additionally, we explored the application potential of the flexible GMR sensor array as both an intelligent and sophisticated magnetoelectronic skin. The flexible GMR sensors were integrated with the magnetic skin; this resulted in the successful demonstration of touchless interactions and the ability to perceive contact pressure. Both of these devices demonstrated strong performance and represent a significant step forward in the development of fully integrated on-skin magnetoelectronics.

## Fabrication

**Fig. 1:**
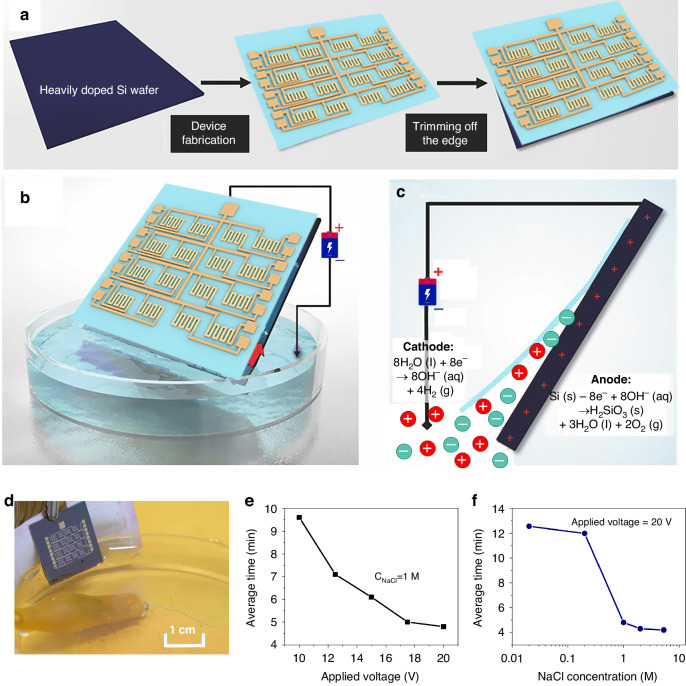
The fabrication of flexible GMR devices. **a** Illustration of a typical electrochemical delamination process. **b** Illustration of a PI-electronic foil preparation on a low-resistance silicon wafer in an NaCl solution. The NaCl solution climbs upward, facilitating the occurrence of reactions. **c** Schematic diagram of the electrochemical reactions at the cathode and anode. **d** Photographs showing the progress in the detachment of a 1.6 cm × 1.6 cm GMR sensor. **e** Average delamination time when applying a voltage ranging from 10 to 20 V; f Average delamination time when the NaCl concentration increases from 0.02 M to 5.43 M (supersaturation)

Figure 1a illustrates a typical electrochemical delamination procedure^34^. Initially, a thin polymer layer is deposited on a heavily-doped silicon wafer, serving as the stiff substrate. Subsequently, GMR devices are fabricated on the upper surface of this film using conventional microfabrication techniques, as depicted in Figure S1 of the Supplementary Materials. Next, the silicon wafer is inclined to contact the surface of the sodium chloride electrolyte aqueous solution using its bottom edge, as shown in Fig. 1b. Notably, the polymer layer around the periphery of the silicon (Si) wafer is gradually removed, ensuring direct contact between the Si wafer’s top surface and the electrolyte solution. Subsequently, positive and negative potentials are applied to the Si wafer and the electrolyte solution, respectively. To optimize the electrochemical processes, the resistivity of the silicon (Si) wafer must be within the range of 0.002 to 0.004 Ω cm. Following this, the positively polarized silicon wafer initiates an anodic reaction, as shown in Fig. 1c:

$${Si}\left(s\right)-8{e}^{-}+8{{OH}}^{-}\left({aq}\right)\to {H}_{2}{{SiO}}_{3}\left(s\right)+3{H}_{2}O\left(l\right)+2{O}_{2}\left(g\right)$$

As a result of an anodic etching process, spaces are created between the polymer and the silicon wafer. During this process, the formation of oxygen bubbles exerts a gentle upward pull, aiding the separation of the two layers. Moreover, the upper electrical components of the thin film are unharmed due to the absence of moisture and dirt. Throughout the process, the ultrathin layer containing the electronic systems detaches successfully from the underlying Si wafer, avoiding significant mechanical or chemical damage and thus ensuring complete and effective delamination. This method demonstrates superiority over other delamination techniques due to its distinctly straightforward, notably rapid, noninvasive, and adaptable nature.

Figure 1d and Movie S1 in the Supplementary Materials illustrate the comprehensive delamination process of a flexible GMR sensor foil. The foil consists of a polyimide substrate measuring 1.6 cm × 1.6 cm with a thickness of 0.97 μm (refer to the step profiler photograph in Figure S2). A detailed description of the fabrication process is provided in the Supporting Information, Note 1. The ultrathin electronic foil was exfoliated from the Si wafer in 4.8 minutes using an applied voltage of 20 V and a 1 M NaCl solution. At the initiation of the delamination process, the electrolyte solution infiltrates the interface between the polyimide and Si along the lateral margins of the Si wafer. As peeling progresses, two fan-shaped domains form at the bottom of the Si wafer and eventually merge. The electronic components positioned at the top remain separate from the solution, while the substrate film undergoes a controlled exfoliation process, ensuring no physical harm. Wrinkling was observed on the film and was attributed to trapped O_2_ bubbles beneath it; due to its ultra-thinness and low bending strength, the film is susceptible to rapid ruffling by slight perturbations. Ultimately, a free-standing film is obtained that is capable of recovering its flatness after being picked up and can be conformably laminated onto target surfaces.

Managing the critical variables in the delamination process has significant importance for the manufacturing of flexible devices. The effects of the applied voltage and NaCl concentration on the delamination of polyimide films from silicon wafers were studied considering the involved electrochemical reactions. Due to the varying delamination rates during processing, these effects were measured based on the time taken to remove an entire film of the same size, as shown in Movie S2. As demonstrated in Fig. 1e, with a constant NaCl electrolyte solution concentration of 1 M and an increase in the applied voltage from 10 V to 20 V, an increase in the average time is observed. With the voltage increase from 10 V to 12 V, an acceleration in the anode reaction and an increase in stacking efficiency are observed. At a voltage of 20 V, the peeling rate progressively increases until reaching a maximum value and then stabilizes. This phenomenon could be attributed to the saturation of NaCl, hindering the peeling process. However, the average delamination time remains relatively short and is typically a few minutes. This rate is significantly faster than that of traditional sacrificial methods, which usually require tens of minutes to several hours. Exfoliation can be accelerated by increasing the NaCl concentration to supersaturation (i.e., 5.43 M at 293 K, 101.325 kPa); this acceleration is likely due to the increased conductivity of the solution enhancing the electrochemical reaction. Due to the anticipated advantages of the rapid delamination and the low cost of the NaCl solution, this process is expected to advance as a method for next-generation large-scale flexible device manufacturing. Figure 1f compares the delamination process across various voltages and NaCl concentrations (0.02 M, 0.2 M, 1 M, 2 M, and 5.43 M). This exfoliation technique demonstrates significant adaptability and provides an efficient method to produce ultrathin electronic systems. The aforementioned electronic system, with its remarkably thin structure, can be seamlessly integrated into human skin or organs. This technological advancement is expected to find applications in the development of future human‒machine interface devices. An ultrathin and flexible giant magnetoresistive sensor array was developed to enable high-resolution pressure detection and facilitate human-computer interactions.

**Fig. 2:**
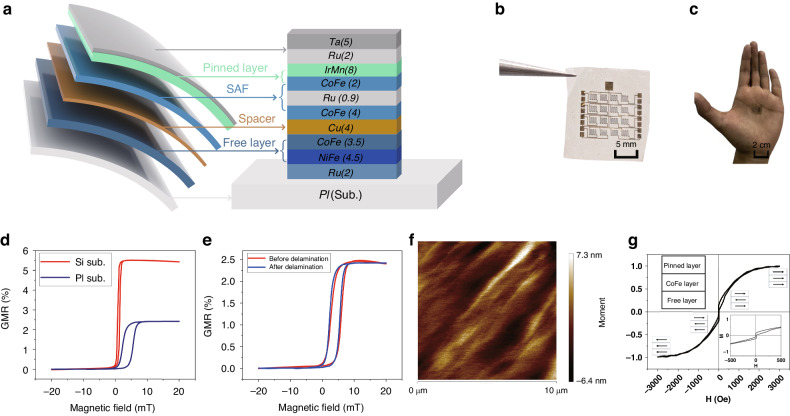
The structure and magnetic properties of flexible GMR devices. **a** Schematic of the GMR spin valve structure deposited on a Si substrate with a synthetic antiferromagnetic layer. **b** Optical photographs of the flexible GMR sensor array. **c** GMR sensor array placed on a human palm. **d** Magnetoresistance curves of a GMR sensor on silicon and PI substrates. **e** Magnetoresistance curves of the GMR sensor before and after delamination. **f** Atomic force microscopy image showing the surface roughness of the spin-coated polyimide on the Si substrates, exhibiting an average roughness of 0.574 nm at 6 different points. **g** Hysteresis loop of the GMR spin valve film

The previously described delamination method enables the direct fabrication of the GMR multilayer structures (Fig. 2a) on polyimide, ensuring compatibility with conventional micro-nanofabrication processes and suitability for large-scale manufacturing. The completed GMR devices are shown in Fig. 2b; the configuration has 16 GMR devices organized in a 4 × 4 array structure with a collective thickness of approximately 0.97 μm. Figure 2c shows the extreme weight and flexibility of the sensor elements, which are capable of withstanding multiple instances of friction and folding.

## Performance

Device testing was conducted on an MR sensor testing platform (TRUTH INSTRUMENTS CO. LTD, PS1DX-MS), and devices with the same dimensions were fabricated on rigid SiO_2_ substrates using identical manufacturing processes. Detailed information regarding the fabrication process is available in the Supplementary Materials, Figure S1. The GMR ratios for the devices on the Si substrates prepared under ambient conditions were observed to reach 5.6%; this is a typical value for artificially synthesized antiferromagnetic layer-based GMR devices^35^. Although the GMR ratio is modest, it exhibits heightened sensitivity to low magnetic fields (10 Oe), particularly compared to other studies^36^, where the effective magnetic field is up to 4 kOe. However, the devices on polyimide substrates exhibited a significant decrease in performance, with the magnetoresistance ratio decreasing from 5.6% to 2.46% and the saturation field increasing to 80 Oe, as illustrated in Fig. 2d. Atomic force microscopy (AFM) was used to assess the surface roughness of the spin-coated polyimide on the Si substrates; these exhibited an average roughness of 0.574 nm, which showed an increase in the roughness of the wafer before spin-coating (0.163 nm), as depicted in Fig. 2f and Figure S3. The analysis indicated that the increased roughness due to the polyimide was likely the primary cause of the performance degradation. Since GMR devices with this structure required stringent control of the substrate quality and deposition parameters, the increased roughness induced by the polyimide significantly impacted the device performance.

Furthermore, tests were conducted on the devices before and after delamination, as illustrated in Fig. 2e. The device curves before and after delamination closely resembled each other, demonstrating a similar magnitude of the GMR. These results indicated that the delamination method employed had a minimal impact on the performance of the GMR devices, further verifying that the performance degradation was primarily caused by the roughness introduced by the polyimide.

**Fig. 3:**
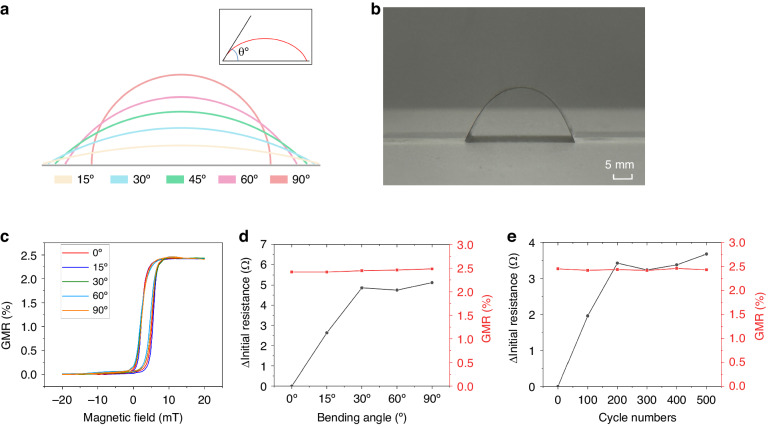
The mechanical properties of flexible GMR devices. **a** Schematic images showing the sample at various bending angles. **b** Optical images of a flexible bending GMR sensor array. **c** Magnetoresistance curves of the GMR sensor for bending angles ranging from 0° to 90°. **d** GMR magnitude (represented by red squares) and resistance change (black dots) as a function of the various bending angles. **e** GMR magnitude (red squares) and resistance change (black dots) as a function of the number of cycles

To evaluate the bending performance of the GMR devices, they were positioned on a specially designed flexible circuit board, enabling simultaneous bending with the circuit board from 0° to 90°, as illustrated in Fig. 3a. Figure 3b shows representative side-view images of the samples in their bent state. The contacts on the GMR devices were connected using conductive silver paint (SPI #5002-AB Silver Paint) and flexed in concert with the flexible circuit board. In Fig. 3c, the GMR ratio consistently remained at 2.46% at any bending angle, independent of the bending state, and the initial resistance of the GMR devices remained stable (approximately 1600 Ω), fluctuating by less than 6 Ω, as shown in Fig. 3d. The minor changes in resistance after bending were likely caused by the bending of the conductive sliver path or the device resistance drift, and the minor changes did not have a significant impact on the device, thereby illustrating the stability of the GMR device. These attributes collectively indicated that the GMR system could be affixed to any part of the human body and remain unaffected by natural skin curvature; thus, this system is particularly valuable for applications in smart skin and biomedical contexts. Furthermore, due to its ultrathin and flexible nature, the GMR system could adhere to any flat surface, providing potential applications in diverse fields, including robotics and electronic power transmission.

Concurrently, repetitive loading experiments were conducted to validate the long-term stability of the flexible GMR devices. The flexible GMR devices were set at a bending angle of 60°, a configuration typically suitable for most skin-related and various field applications. These repetitive loading experiments were carried out using a mechanical stage. As shown in Fig. 3e, even after more than 500 loading cycles, the GMR ratio remained at 2.46%, and the initial resistance of the multilayer films minimally changed (varying by less than 4 Ω); moreover, this change did not significantly impact the device performance. This long-term stability attested to the durability of flexible GMR devices; thus, these devices provide a robust foundation for diverse applications.

**Fig. 4:**
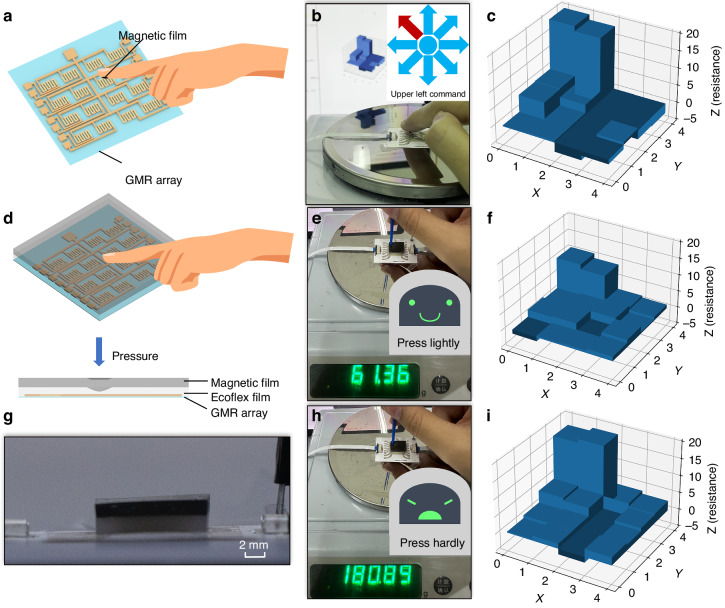
The contact and non-contact applications of flexible GMR devices. **a** Schematic image showing the touchless interactions using the flexible GMR sensor array. **b** Optical image of the flexible GMR sensor array and magnetic skin on a human fingertip. **c** Magnetic response of the GMR sensor array when a finger approaches the top left corner. **d** Schematic images showing the pressure sensing using the flexible GMR sensor array. **e**, **h** Optical images of the flexible GMR sensor array and magnetic skin when pressures of 61.36 g and 180.89 g are applied. **g** Optical side images of the pressure sensing installation. **f**, **i** Magnetic response when pressures of 61.36 g and 180.89 g are applied

The application potential of the flexible GMR sensor array as an intelligent magnetoelectronic skin was explored. Initially, a magnetic skin was fabricated that served as a flexible magnet capable of generating a magnetic field. The magnetic skin could be seamlessly integrated with the flexible GMR array, enabling the GMR devices to sense the magnetic field generated by the magnetic skin. The fabrication process for the magnetic skin is detailed in Figure S4. A 75 wt% magnetic skin, with a radius of 0.8 mm and a thickness of 1 mm, was used; it adhered to a finger near the flexible GMR array. As illustrated in Fig. 4a, the flexible GMR sensor array could respond to the magnetic skin on the fingertip, displaying changes in the surrounding magnetic field, as shown in Fig. 4c; this could act as an ‘upper left’ command in Fig. 4b for a noncontact human‒machine interface. Placing this combination on the human body could endow individuals with an additional sense, known as magnetoreception, which is not naturally possessed.

Subsequently, the flexible GMR array was applied to high spatial resolution pressure sensing, as demonstrated in Movie S3. The magnetic skin and the flexible GMR array were attached with a layer of pure Ecoflex in between, as shown in Fig. 4d (Ecoflex thickness: 2.2 mm, magnetic skin thickness: 1.8 mm, as depicted in Fig. 4g). The magnetic film used for pressure sensing had a magnetic powder content of 50 wt%, and the remanence generated was approximately 5 mT; this value falls within the working range of GMR devices. Additionally, the film possessed a relatively large elastic modulus and was soft. When pressure was applied, the magnetic skin underwent deformation, leading to changes in the magnetic field sensed by the flexible GMR sensor array. The magnetic field output varied in accordance with the magnitude of the applied pressure. The finite element simulation of mechanical deformation under pressure and changes in magnetic field can be found in Figure S7. As demonstrated in Fig. 4e, when a force of 61.36 g was applied in the top-left corner, the output of the flexible GMR devices reached a maximum change of 9.89 Ω, as shown in Fig. 4f. In contrast, when a force of 180.89 g was applied in the same corner, as depicted in Fig. 4h, the output reached a maximum change of 16.46 Ω, as shown in Fig. 4i. This intelligent magnetoelectronic skin provides high spatial resolution (GMR device spacing: 2 mm) and has diverse applications. For instance, it can be used on the human body for multisite pressure, particularly in patients at risk for pressure injuries, or applied to robots to provide high-resolution pressure sensing capabilities and human-like functionality.

**Fig. 5:**
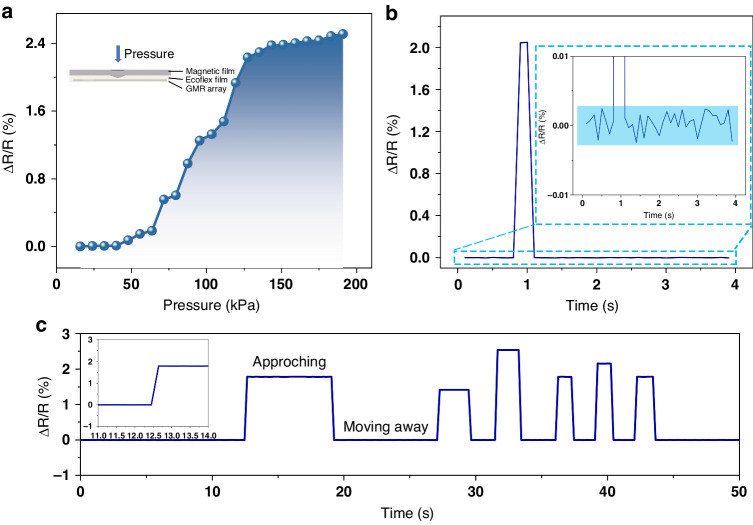
The practical performance of flexible GMR devices in applications. **a** Graph of magnetoresistance ratio variation with pressure. **b** noise level in the pressure sensing. **c** signal variations when the magnetic skin approaches or moves away from the magnetic sensor

Finally, we characterized the relationship between the pressure and the changes in magnetoresistance. As shown in Fig. 5a, as the pressure increases, the deformation between the magnetic and nonmagnetic layers increases. The magnetic part approaches the flexible GMR array at the bottom, and the magnetic field experienced by the GMR devices also increases; this results in increased resistance changes. Additionally, the relationship of the GMR device with increasing pressure is similar to that of the magnetoresistance curve. The increase is relatively slow under low pressure, and the change in resistance sharply increases with increasing pressure. This result is consistent with the higher sensitivity between 1 and 6 mT on the magnetoresistance curve. As the pressure continues to increase, the change in the magnetoresistance ratio tends to slow; this is related to the saturation of the magnetoresistance device. Moreover, both the pressure measurement range and sensitivity can be modulated by altering the thickness of the Ecoflex layer and the magnetic skin layer; thus, further experimentation is needed for validation.

Subsequently, the noise levels of the magnetoresistive response was also investigated. As shown in Fig. 5b, this sensitive unit demonstrated an excellent performance in terms of the signal-to-noise ratio. In the absence of an external force, changes in the magnetoresistance ratio were minimal and did not exceed 0.005%. However, once an external force was applied, the change in the signal significantly increased, with a signal-to-noise ratio greater than 400. Similarly, tests were conducted to evaluate the output response of a single sensor to the magnetic skin; this demonstrated its real-time responsiveness, as shown in Supplementary Materials Movie S4. The sampling time was set to 0.2 s, enabling the sensor exhibited near real-time responsiveness to the magnetic skin, as shown in Fig. 5c.

## Conclusion

In this study, a simple and rapid method for the electrochemical delamination of flexible electronic systems from ultrathin films was developed. Using this method, the first flexible GMR sensor array with a spin-valve structure exhibiting increased sensitivity to small magnetic fields was fabricated. Since microelectronic fabrication processes can be directly applied to silicon wafers, lithographic patterning of magnetoelectronic nanostructures on ultrathin PI foils was successfully achieved, and the separated electronic systems remained intact and undamaged.

The magnetoelectronic skin was exceptionally lightweight and conformable on any surface and could bend up to 90 degrees without any degradation in performance. The significant potential of this technology in applications such as smart skin and medical implants was demonstrated. Noncontact interaction applications were shown, where placing the magnetic skin on the fingertips enabled the execution of various commands by moving the fingers. Additionally, high spatial resolution pressure sensing was demonstrated by stacking Ecoflex and the magnetic skin on a flexible GMR array, thereby enabling the detection of the applied pressure. Furthermore, the pressure measurement range and sensitivity could be modulated by altering the thickness of the Ecoflex and magnetic skin layers.

Future work will concentrate on optimizing the GMR device performance, minimizing hysteresis, and exploring the relationships among the pressure measurement range, the sensitivity and thicknesses of the Ecoflex, and the magnetic skin layers. Moreover, optimization of the electrical and mechanical interfaces with other electronic components, such as wireless readouts and remote sensing, will be pursued. The integration of magnetoelectronics with ultrathin functional devices, including solar cells, transistors, and temperature sensor arrays, will facilitate the development of integrated electronic skins with enhanced functionality.

### Supplementary information

Supplementary Materials

Movie S1. Electrochemical delamination process

Movie S2. Electrochemical delamination processes under different conditions: (1) Voltage: 20 V, NaCl concentration: 1 M; (2) Voltage: 10 V, NaCl concentration: 1 M; (3) Voltage: 20 V, NaCl concentration: 0.2 M

Movie S3. Illustration of the pressure sensing using a flexible GMR sensor array

Movie S4. Illustration of real-time performance in a noncontact human‒machine interface using a flexible GMR sensor array
