# Supplementary material for: An ultrathin, rapidly fabricated, flexible giant magnetoresistive electronic skin
Source: Microsyst Nanoeng. 2024 Aug 12;10:109. doi: 10.1038/s41378-024-00716-2 (PMC11319584; doi:10.1038/s41378-024-00716-2)
Supplement: Supplementary file 1 — Supplementary Materials [file 41378_2024_716_MOESM1_ESM.pdf]

Supporting Information

An ultrathin, rapidly fabricated, flexible giant magnetoresistive electronic skin

Junjie Zhang[1,2], *Zhenhu Jin*[1,2,*] *,Guangyuan Chen*[1] *and Jiamin Chen*[1,2,3,*]

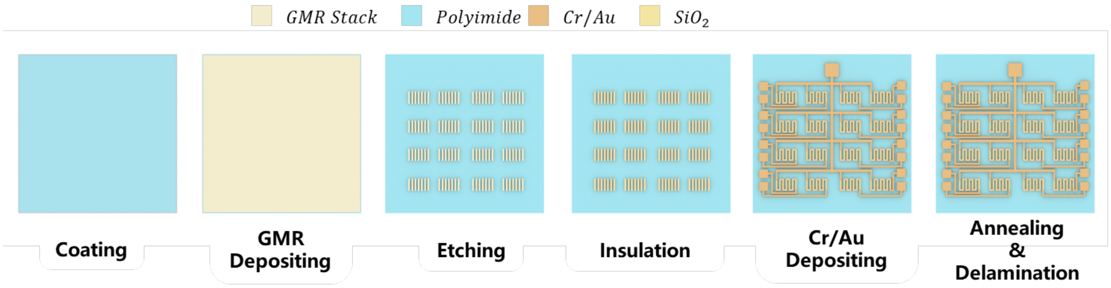

Figure S1. The manufacturing process of GMR devices.

The manufacturing process of GMR devices is as follows:

1. Spin-coating of PI Film:

- A 4-inch silicon wafer was prepared using spin coating to deposit a polyimide (PI) film, with a spinning speed set at 1200 rpm.

2. Multilayer Film Deposition:

- After curing, a multilayer film was deposited using room-temperature magnetron sputtering deposition (ULVAC, QAM-4W). The film consisted of the following layers: Ru (2)/$Ni_{81}Fe_{19}$(4.5)/ $Co_{75}Fe_{25}$(3.5)/ Cu (4)/ $Co_{75}Fe_{25}$(4)/ Ru (0.9)/ $Co_{75}Fe_{25}$(2)/ $Ir_{22}Mn_{78}$(8)/ Ru (2)/ Ta (5).

3. Patterning GMR Devices:

- Photolithography techniques were employed to pattern the GMR devices. The dimensions of each GMR device were 100 µm x 1000 µm. Seven of these devices were serially connected in a folded configuration. Ion beam etching (Advance, LKJ-1A-150) was utilized to etch the GMR strips.

4. Deposition of Dielectric Layers:

- Chemical vapor deposition (CVD) methods were used to deposit dielectric layers of silicon dioxide (SiO2) and silicon nitride (SiNx), each with a thickness of 150 nm. These layers served as electrical isolation. Photolithography techniques were also employed to create openings for electrode connections.

5. Electrode Definition:

- Photolithography techniques were used to define the electrode shapes. Electrodes were deposited in the following order: 15 nm of tantalum (Ta) followed by 100 nm of gold (Au).

6. Annealing:

- All GMR devices were annealed in a vacuum at 180°C for 1 hour under a bias magnetic field of 10,000 Oe. During the annealing process, the GMR stripes were oriented perpendicular to the direction of the applied exchange bias field. This annealing step was carried out in a Vacuum Magnetic Annealing Furnace (Model F800-35/EM7, East Changing Technologies, China).

7. Electrochemical Delamination:

- Finally, an electrochemical delamination method was employed to detach the flexible GMR devices.

This comprehensive process outlines the fabrication of the GMR devices, incorporating various deposition techniques and lithography methods to achieve the desired structure and properties.

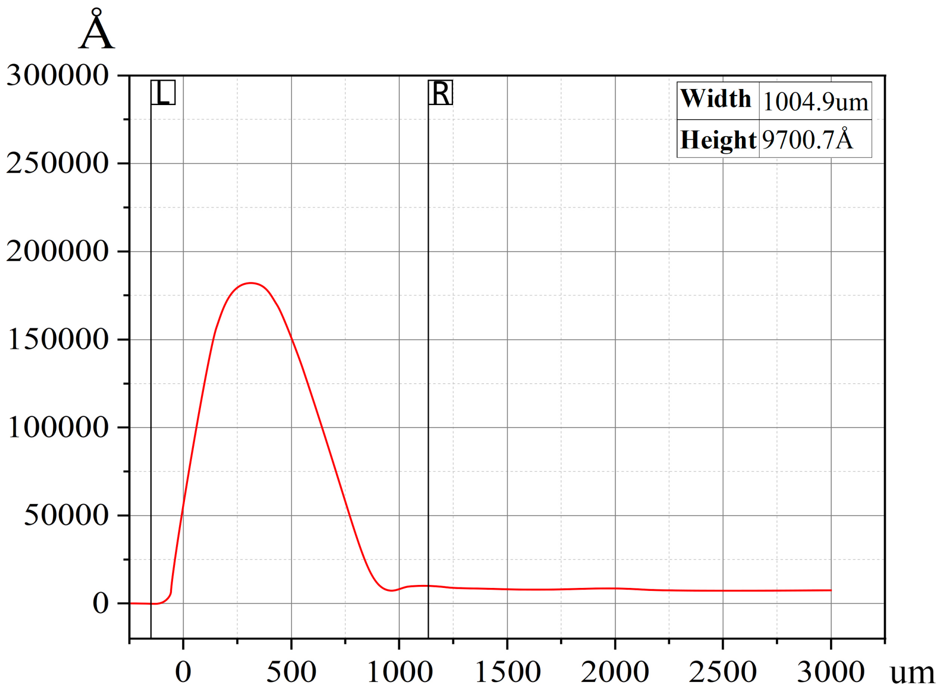

Figure S2. The step profiler photographs of the PI foil.

The figure depicts the surface profile of a 3-micrometer scan along the edge of the PI film. There is a protrusion at the edge of the PI foil caused by cutting, which can be disregarded. The remaining portion can be used to calculate the thickness, with the dashed line corresponding to a thickness of 0.97 µm.

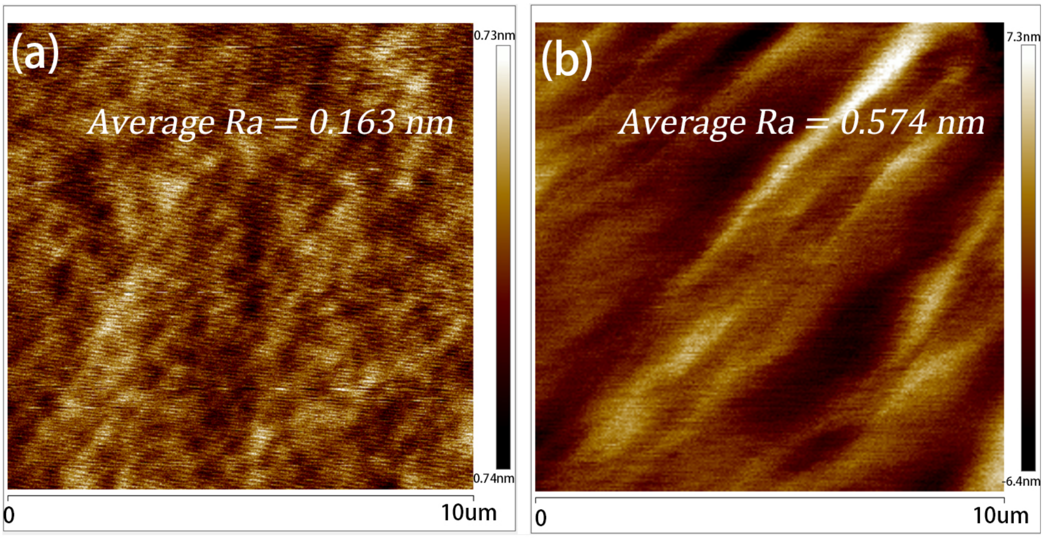

Figure S3: Atomic force microscope images of the Si substrate before and after spin-coating with polyimide are presented, providing a comparison of the roughness before and after the spin-coating process. The average roughness measured across six points on the wafer was found to be 0.163 nm (Figure S3(a)), after spin-coating with polyimide, the average roughness increased to 0.574 nm (Figure S3(b)).

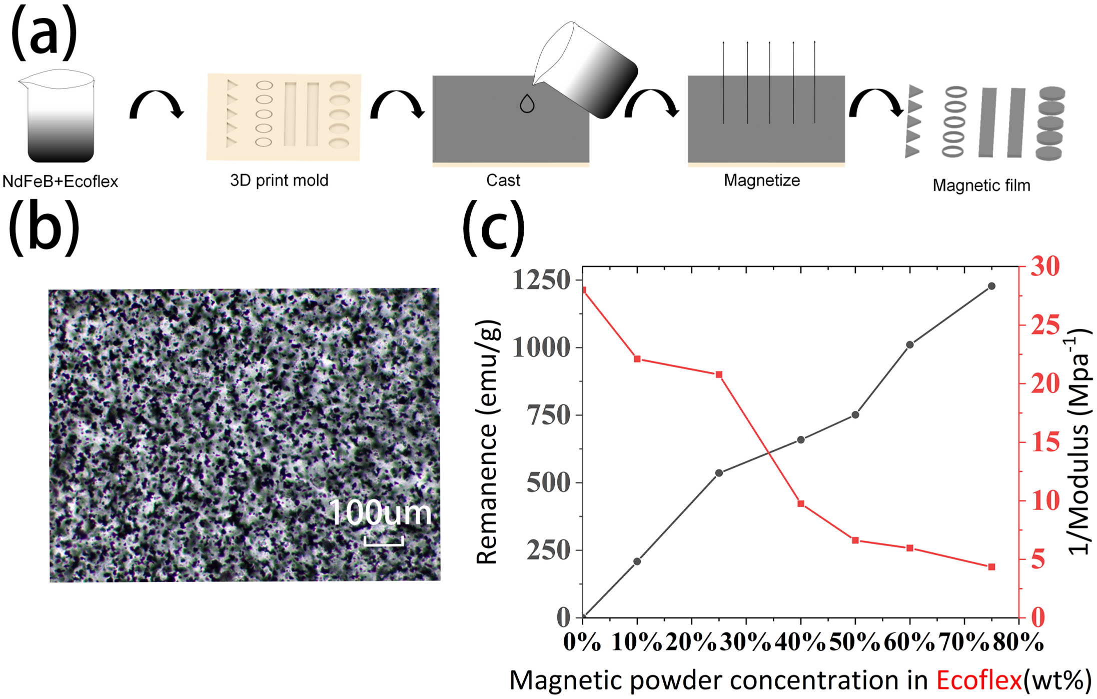

Figure S4. (a) Fabrication process of the magnetic skin; (b) T Scanning electron microscope characterization of the magnetic skin; (c) Taking Ecoflex as elastic matrix, the characterization of the modulus of elasticity and remanent magnetizations versus different content of the magnetic powder in the magnetic skin.

The manufacturing process of magnetic skin: Magnetic skin is made of elastic matrix and permanent magnetic powder. The elastic matrix materials is Ecoflex(Smooth-on, 00-50), the magnetic powder can be NdFeB. Figure S1 (a) illustrates the fabrication process: first, the composite material is prepared by mixing the elastic matrix and permanent magnet powder. And then the 3D printing mold is prepared with the required shape and size. The mixture is cast into the mold, and the casting blade is used for planarization. After curing, the magnetic skin is magnetized along the out of plane direction, and finally the composite material is released from the mold to make the magnetic skin. The magnetic skin is magnetized using 2 T external magnetic field for 1 hour. Figure 1 (b) shows the scanning electron microscope (SEM) of the magnetic skin, in which the black particles are permanent magnet powder and the light part is elastic matrix. The materials of the elastic matrix and the magnetic powder, as well as the mixing ratio, will be discussed in Figure 1 (c) to illustrate the influence on the properties of the magnetic skin.

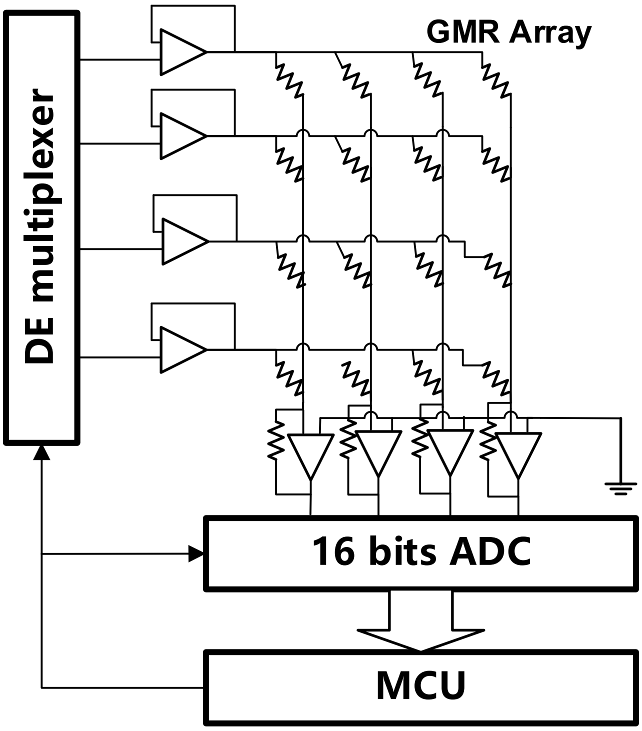

Figure S5. The signal processing circuit of GMR devices.

The diagram illustrates the readout process for an array of resistors. Initially, rows to be read are selected using a multiplexer (RS2251XTSS16) and subjected to a working voltage of 1.25V provided by a voltage regulator (REF3012AIDBZR). Subsequently, the circuit interfaces with a GMR sensor array and an operational amplifier (TLV2374Q), with a reference resistor of 1.5kΩ to match the resistance of the GMR device. The operational amplifier is connected to a 16-bit analog-to-digital converter (AD7606). Finally, data is transmitted to a computer via serial communication using a microcontroller (STM32F407VET6) to display changes in the magnetic field above the GMR sensor array.

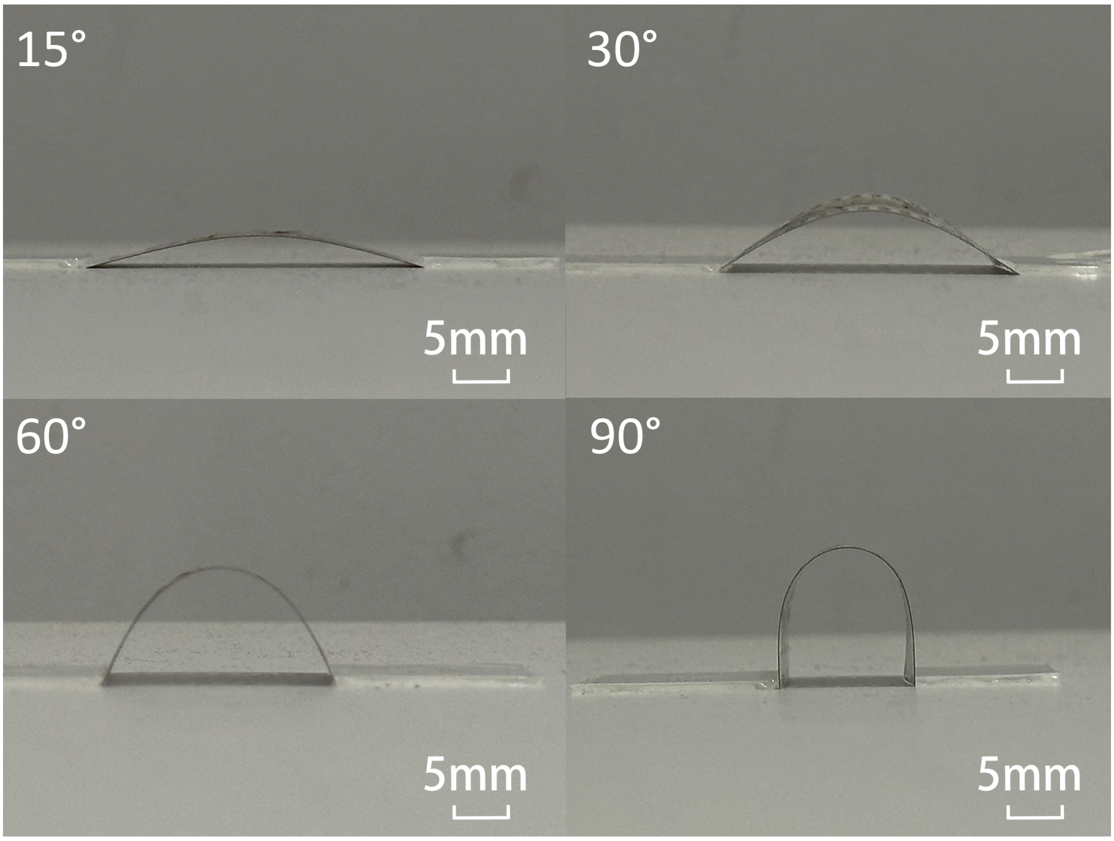

Figure S6. Bending at Different Bending Angles: Photographs of bending at various bending angles, specifically 15°, 30°, 60°, and 90°.

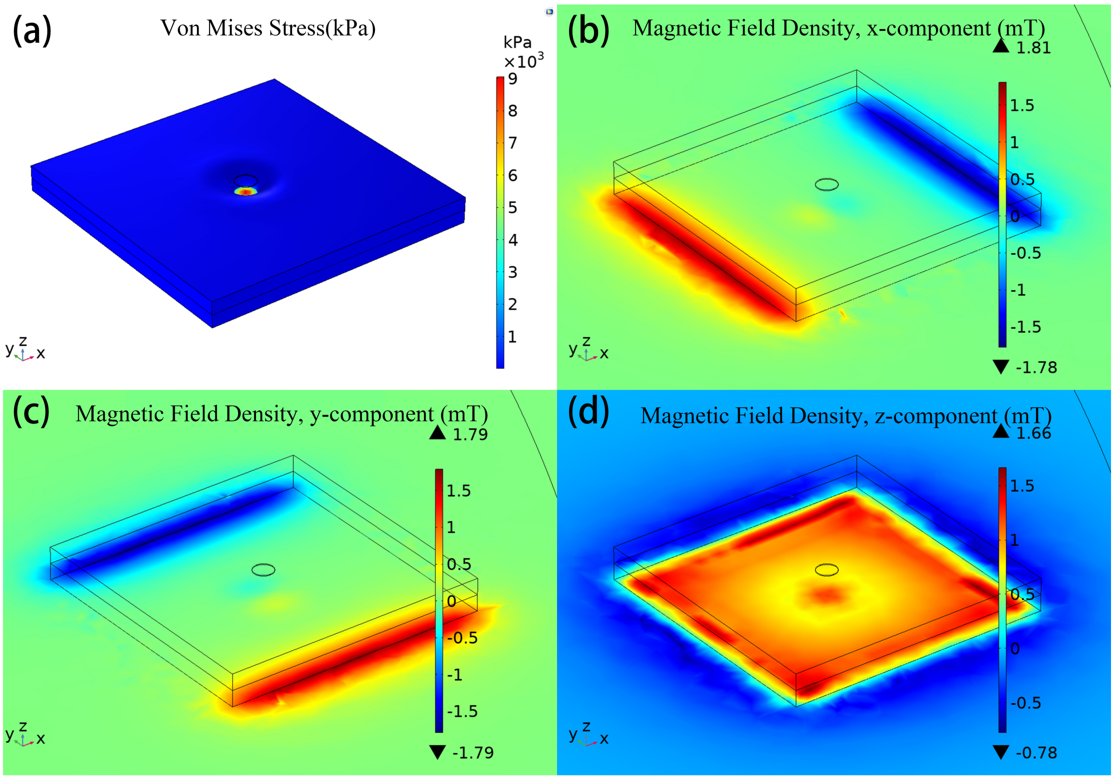

Figure S7. Finite element simulation of piezomagnetic electronic skin pressure sensing: (a) Stress simulation after applying force; (b, c, d) Magnetic field distribution in the x, y, z direction after applying force.

A pressure sensing unit was constructed using Comsol and subjected to finite element simulation. The unit is composed of a 1.8 mm magnetic layer and a 2.2 mm non-magnetic layer, as same in the manuscript. The area where force is applied is set as a circle with a radius of 1 mm. The presence of the non-magnetic layer helps to expand the range of force detection. When a force of 0.4N is applied, the stress change of the pressure sensing unit is as shown in Figure S7(a), and deformation can be observed in the area where the force is applied due to the soft characteristics of two layers. Based on this deformation, a magnetic field simulation was further added to calculate the magnetic field distribution on the bottom surface under this deformation. Figures 6-S7(a, b, c) show the magnetic field distribution in the x, y, and z directions, respectively.

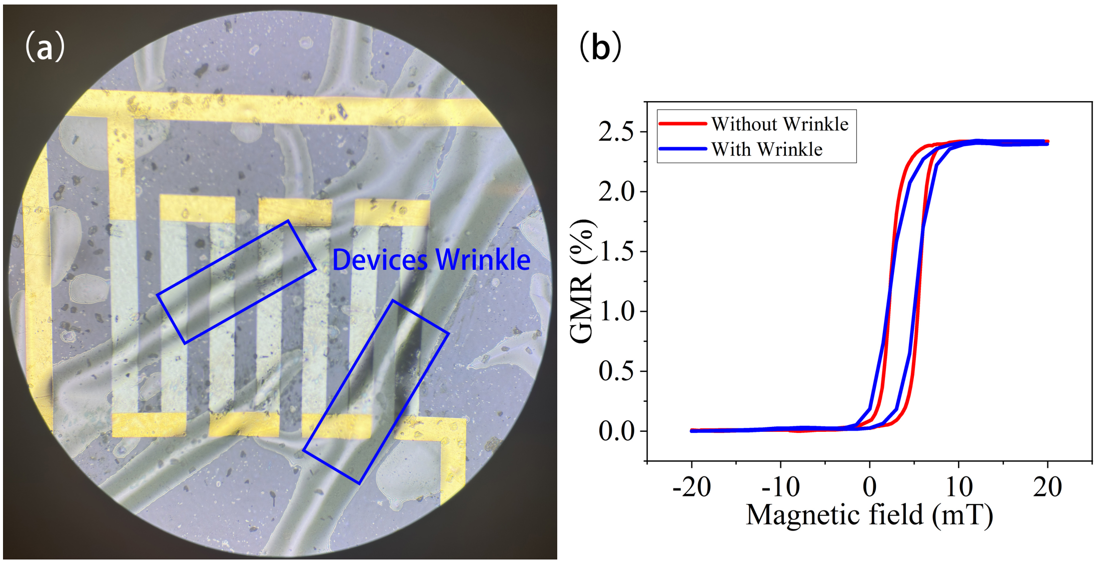

Figure S8. (a)The wrinkles in the flexible GMR sensors; (b)The comparison of magnetoresistance curves between devices with and without wrinkles.

We also evaluated the performance of devices with bending radius on the millimeter level. During the testing phase, the device was not kept entirely flat, as shown in Figure. S8(a). Wrinkles appeared on the array devices, causing the device to possess a bending radius at the micrometer level (we regret that we do not have the appropriate equipment to measure this specific value). Subsequently, the magnetoresistance curve of the device was tested and compared with a wrinkle-free device, as shown in Figure. S8(b). The device with wrinkles exhibited a magnetoresistance curve similar to that of a normal device with slight differences in effective fields and sensitivity, and the magnetoresistance ratio was maintained at around 2.46%. This further validates that the flexible magnetic sensor can maintain normal performance even under some extreme conditions.

Supplementary Note 1: Electrochemical Etching-Assisted Delamination:, A polyimide (PI) film was fabricated using spin coating techniques at a speed of 1200 rpm on a 4-inch N-type silicon wafer with a resistivity of 0.002-0.004 Ω and a thickness of 500±10 μm. Subsequently, various concentrations of NaCl solution (0.02 M, 0.2 M, 1 M, 2 M, and 5.43 M) were prepared in water, and the sectioned silicon wafer (1.6 cm × 1.6 cm) was mounted using clamping equipment with its device layer oriented upwards, ensuring the lower edge of the wafer was in direct contact with the NaCl solution. The silicon wafer was subjected to a positive polarization, while the NaCl solution received a negative polarization. As the reaction progressed, the PI film, incorporating the GMR devices, successfully detached from the silicon surface. The rate of separation was quantified by measuring the time required for complete detachment.

Supplementary Note 2: GMR multilayers on ultrathin Polyimide: PI foil (RDPI 0.2) was deposited onto a 4-inch silicon wafer using spin-coating techniques at 1500 rpm for 2 hours, followed by curing in an oven at 120°C. The resulting surface roughness of the foil measured approximately 0.574 nm. Following the curing process, a multilayer film consisting of Ru (2)/$Ni_{81}Fe_{19}$(4.5)/ $Co_{75}Fe_{25}$(3.5)/ Cu (4)/ $Co_{75}Fe_{25}$(4)/ Ru (0.9)/ $Co_{75}Fe_{25}$(2)/ $Ir_{22}Mn_{78}$(8)/ Ru (2)/ Ta (5)) was fabricated using room-temperature magnetron sputtering deposition (ULVAC QAM-4W), with a base pressure of 5×10^{-6} Pa and an Ar sputtering pressure of 5×10^{-1} Pa, setting the deposition rates to 0.05222 nm/s (80 W) for Ru, 0.06617 nm/s (80 W) for NiFe, 0.03932 nm/s

(60 W) for CoFe, 0.01111 nm/s (80 W) for Cu, 0.06406 nm/s (80 W) for IrMn, and 0.05882 nm/s (80 W) for Ta.

Supplementary Note 3: Magnetoresistive Responses: Testing of the devices was performed on an MR sensor testing platform (TRUTH INSTRUMENTS CO.LTD, PS1DX-MS), utilizing a bipolar power supply to generate an in-plane magnetic field via an electromagnetic coil. The magnetoresistive properties of the GMR devices were analyzed in both bent and flat configurations, using a 4-wire measurement setup. The accuracy of magnetic field detection was found to exceed 0.01 mT, and the maximum strength of the magnetic field reached 500 Gs. For the experimental procedures, currents approximately 1 mA were supplied using a Keithley 6221 current source, and voltage measurements were obtained using a Keithley 2182A nanovoltmeter.

Supplementary Note 4: GMR bending performance: The bending performance of GMR devices was evaluated through static mechanical bending tests utilizing a custom-designed bending apparatus. Flexible GMR devices were mounted on a flexible circuit, and their electrode contact points were connected using conductive silver paint. The GMR devices were affixed to glass slides, with the spacing between the slides dictating the bending angle of the devices. Magnetoresistive performance measurements were conducted in both bent and flat states. Repetitive loading experiments were carried out using a programmable mechanical stage. By setting the distance between the mechanical arms to repeatedly move, the GMR devices underwent repeated bending between 0° and 60° angles. Following every one hundred cycles of repetitive loading, the magnetoresistive performance of the GMR devices was evaluated and documented.

Movie S1. An electrochemical delamination process.

Movie S2. Electrochemical delamination processes under different conditions: (1) Voltage: 20 V, NaCl concentration:1 M; (2) Voltage:10 V, NaCl concentration:1 M; (3) Voltage:20 V, NaCl concentration:0.2 M.

Movie S3. Illustration of pressure sensing using the flexible GMR sensor array.

Movie S4. Illustration of real-time performance in non-contact human-machine interface using the flexible GMR sensor array.
